# Editorial: Immune response in tuberculosis with comorbidities or coinfections

**DOI:** 10.3389/fimmu.2025.1665988

**Published:** 2025-08-12

**Authors:** Adriano Gomes-Silva, Subash Babu, Valeria Cavalcanti Rolla

**Affiliations:** ^1^ Laboratório de Pesquisa Clínica em Micobacterioses, Instituto Nacional de Infectologia Evandro Chagas, FIOCRUZ, Rio de Janeiro, Brazil; ^2^ Department of ICER, National Institutes of Health-National Institute of Allergy and Infectious Diseases-International Center for Excellence in Research, Chennai, India; ^3^ Laboratory of Parasitic Diseases, National Institutes of Allergy and Infectious Diseases, National Institutes of Health, Bethesda, MD, United States

**Keywords:** tuberculosis, immune response, coinfection, comorbidities, helminth

Tuberculosis (TB) remains a leading cause of infectious disease mortality globally. Its pathogenesis is now understood to be heavily shaped by immunometabolic perturbations and host comorbidities. In this context, the ten articles summarized in this Research Topic collectively deepen our understanding of how metabolic disorders, immune-mediated diseases, helminth infections, and viral coinfections converge to modulate TB immune response, whose profiles may be associated with disease susceptibility or post-treatment outcomes.

Several studies in this Research Topic illuminate the complex interplay between diabetes mellitus (DM) and TB. Araujo-Pereira et al. offer a comprehensive overview of TB-DM comorbidity, identifying chronic systemic inflammation and impaired immune regulation as pivotal mechanisms fueling susceptibility and complicating treatment. Complementing this, Ssekamatte et al. show how type 2 diabetes alters *Mycobacterium tuberculosis* (Mtb)-specific CD4^+^ and CD8^+^ T-cell phenotypes, reducing interferon gamma production and this occurs via an increasing of programmed cell death protein-1 expression, underscoring the impact of functional immune exhaustion in individuals with latent Mtb infection (LTBI). Rajamanickam et al. further demonstrate that TB with prediabetes is characterized by exacerbated profiles of pro-inflammatory cytokine and chemokine, linking immune dysfunction to glycemic dysregulation. Additionally, Ranaivomanana et al. reveal that DM skews longitudinal treatment monitoring biomarkers—specifically, the monocyte-to-lymphocyte ratio and the release of interferon gamma produced by T-cells specific to Mtb antigens—potentially limiting their interpretive value in diabetic TB patients.

A critical implication of these findings is the pressing need for immunomonitoring tools and personalized treatment strategies for TB-DM patients, especially in low-resource settings where this comorbidity is on the rise.

Beyond metabolic disorders, anemia emerges as an underappreciated but potent modulator of TB outcomes. Dasan et al. demonstrate that anemic TB patients experience higher bacterial burdens, more extensive lung pathology, and worse treatment outcomes. These effects are mediated through an imbalanced cytokine milieu favoring pro-fibrotic and inflammatory pathways. This adds a new dimension to TB management, suggesting that addressing anemia could serve as an adjunctive intervention.

The immune landscape is further complicated by helminth coinfections, as shown by Pushpamithran and Blomgran. Their data indicates that *Ascaris lumbricoides* antigen exposure reprograms macrophage-derived extracellular vesicles (EVs) to enhance Mtb control while dampening Interleukin-1 betaproduction, via microRNA-mediated modulation of Phosphoinositide 3-kinase/Akt pathway and Mitogen-Activated Protein Kinase pathways. This illustrates the immunoregulatory potential of helminths and the therapeutic promise of EVs as immunomodulators in TB.

Autoimmune conditions and their treatments also significantly affect TB immunobiology. Farroni et al. report that patients with immune-mediated inflammatory diseases (IMID), such as rheumatoid arthritis, retain intact Mtb-specific immune responses production, and this occurs via via *in vitro* bacterial control, despite their underlying immune dysfunction and immunosuppressive therapies. However, Picchianti-Diamanti et al. warn that immunomodulatory biologics, particularly tumor necrosis factor inhibitors and Janus tyrosine kinase inhibitors, significantly increase TB risk, underscoring the importance of rigorous LTBI screening and prophylaxis in these populations. The discrepancy between preserved immune function and elevated TB risk raises important questions about the balance between systemic and compartmentalized immunity in the context of immunosuppressive biologic therapies.

Although the relationship between other viral infections and TB is not as strong as that of human immunodeficiency virus, some can affect immunity and increase the risk of developing the disease or worsening its course. However, due to its recent circulation in the population, little is known about the impact of Severe Acute Respiratory Syndrome Coronavirus 2 on patients infected with Mtb Peña-Bates et al. show that individuals with LTBI and mild coronavirus disease 2019 (Covid-19) exhibit enhanced CD8^+^ T cell cytotoxicity, mitochondrial stability, and attenuated pro-inflammatory cytokine secretion compared to Covid-19-only patients, suggesting a potentially immune modulation conditioning by LTBI. Meanwhile, Kameni et al. reveal that polymorphisms in the angiotensin-converting enzyme 2 gene influence cytokine responses in TB-Covid-19 co-infection, suggesting that host genetics modulate disease severity in co-infected individuals.

Together, these studies underscore the necessity for an integrative view of TB pathogenesis, one that incorporates immunogenetics, comorbidity profiles, and host-pathogen-microbiome interactions. They also call for the development of stratified treatment and monitoring protocols that reflect the heterogeneity of TB hosts in real-world clinical settings.

However, gaps remain. While robust in design, several studies rely on small cohorts, lack longitudinal validation or confined to geographically distinct locations. Functional studies linking cytokine signatures to bacterial clearance or tissue pathology are needed to move from correlation to causation. Moreover, the interplay between metabolic control (e.g., glycated hemoglobin levels) and immune trajectory during TB therapy is insufficiently characterized, limiting translational applications.

In conclusion, the convergence of TB with metabolic, autoimmune, parasitic, and viral comorbidities demands a multidimensional approach to research and care. Immunological profiling, coupled with genetic screening and clinical biomarkers, holds promise for identifying vulnerable subpopulations and optimizing TB control strategies. As these findings integrate into practice, they may finally tilt the balance in the global fight against TB.

